# Health, equity and the post-2015 agenda: raising the voices of marginalized communities

**DOI:** 10.1186/s12939-014-0082-6

**Published:** 2014-10-10

**Authors:** Ana Lorena Ruano, Eric A Friedman, Peter S Hill

**Affiliations:** Center for International Health, University of Bergen, Norway/Centro de Estudios para la Equidad y Gobernanza en los Sistemas de Salud, Guatemala City, Guatemala; O’Neill Institute for National and Global Health Law, Georgetown, Guyana; School of Population Health, The University of Queensland, Brisbane, Australia

**Keywords:** MDGs, SDGs, Post-2015 development agenda, Community participation, Go4Health, Right to Health, Go4Health

## Abstract

In September 2012 the United Nations (UN) initiated a process that would extend and enhance the unfinished agenda of the Millennium Development Goals (MDGs), integrating a new vision for sustainable development beyond the year 2015. The initial consultation phase has been completed, with the UN and partner organizations undertaking eleven thematic consultations, including one on health. It is in this context that the European Commission (EC) has tasked the research consortium Goals and Governance for Global Health (Go4Health) with providing recommendations for the post-2015 health-related development goals and including voices that are routinely excluded from health-related decision-making processes. This has not been an easy task. It has led us to question how to define marginalization, how to access marginalized communities, as well as how community members could provide informed consent. The context of the communities we worked with was far removed from the reality of the post-2015 debates, where the MDGs and the new goals are remote and abstract, and where the promise of immediate benefit from participation could not be assured. Given the social, historical, cultural, ethnic and geographical diversity of our chosen community partners, and the diversity of their lived experiences, could their unique situations be generalized in ways that could influence the global debate? In this special issue, we have tried to explore the uniqueness and the commonalities of the issues and barriers that marginalized communities face all over the globe, and present them in individual papers that, together, provide a nuanced and complex picture of the challenges that face the post-2015 health-related agenda setting-process.

In September 2012 the United Nations (UN) initiated a process that would extend and enhance the unfinished agenda of the Millennium Development Goals (MDGs), integrating a new vision for sustainable development beyond the year 2015. The initial consultation phase has been completed, with the UN and partner organizations undertaking eleven thematic consultations, including one on health. It is in this context that the European Commission (EC) has tasked the research consortium Goals and Governance for Global Health (Go4Health) with providing recommendations for the post-2015 health-related development goals. This is to be done through striking a balance between horizontal and vertical approaches to healthcare, through establishing an improved system for global health innovation and through developing measurable, achievable and sustainable goals that reflect the role that both developed and developing countries can play.

As a way to broaden participation and to include more voices in the developing of the new goals, close to a hundred countries started a conversation around the future of their social and economic development processes. Through participation in a universal web-based consultation, hundreds of thousands of people were able to contribute to the shaping of the ‘world we want’ [1]. That process concluded with the report of the High-Level Panel of Eminent Persons on the Post-2015 Development Agenda [2], and responsibility for the development of the Sustainable Development Goals has now passed to the member states through the Open Working Group process. Despite these efforts, large portions of the population were not able to participate, and the issue of how to include the voices of marginalized communities from around the world remains unclear. How can this process represent the poor, the indigenous, the geographically isolated, older and younger persons, minorities and people living with disabilities?

Go4Health is a collaboration between academic and civil society organizations, with funding from the EC, the Australian National Health and Medical Research Council and Canada, and includes 13 research partners from Asia, Africa, Australia, Europe and North and Latin America [3]. In this special issue of the International Journal for Equity in Health, we present the findings from the community consultations carried out with marginalized communities in Africa, Latin America, Australia and the Pacific region. We expect to present findings from the consultations in Asia in the near future. Our aim was to find and raise voices that are routinely excluded from health-related decision-making processes.

Carrying out these consultations has not been an easy task, and we have encountered questions regarding how to define marginalization, how to access marginalized communities, as well as how community members could provide informed consent. This happened in contexts where the reality of the post-2015 debates was remote and abstract, and where the promise of immediate benefit could not be assured. Finally, given the social, historical, cultural, ethnic and geographical diversity of our chosen community partners, and the diversity of their lived experiences, could their unique situations be generalized in ways that could influence the global debate? [4] We have tried to explore the uniqueness and the commonalities of the issues and barriers that marginalized communities face all over the globe, and present them in individual papers that, together, provide a nuanced and complex picture of the challenges that face the post-2015 health-related agenda setting-process.

In rural Guatemala, health was understood in terms of positive relationships with the environment, with neighbours, with friends and family. However, the relationships these Guatemalan communities had with staff in public health facilities was characterized by racism, rudeness and high levels of discrimination that manifested itself in poor quality services and providers that lack empathy and commitment. Yet, despite this treatment, which is often grounded in ethnic prejudice, rural Guatemalan communities were not deterred from their values. They had a compelling message for their councils, for their government, for the global community: “a strong health system requires building on values such as fairness, trust and legitimacy” and they want to be a part of its development and governance [5].

Youth in the Pacific island state of Vanuatu are directly threatened by climate change. As in Guatemala [5], they have a holistic vision of what health is: being healthy is being free from stress and to be able to enjoy a rich family and community life while feeling protected from the changing island environment [6]. However, the isolation of the people from Vanuatu has not protected them from the shadow of globalization: of alcohol and drug abuse, of a changing diet where the products of their own seas and gardens have been traded for processed foods that are high in salt, sugar and fat, of threats to their health today and in the long run. The youth look to the global community for support, but recognise that health is ‘everybody’s responsibility’. While the government and the global community are part of the solution, the capacity for change also lies within their own hands.

That sense of youthful optimism, however, was not part of what older persons and disabled communities in rural Uganda reported [7]. Their perceptions were of lives marginalized even among the marginalized: neglected in their own communities, discriminated against when seeking health care, and unable to access services that addressed their specific needs. Furthermore, care for the disabled and older persons in rural communities is not a priority in a state that struggles to provide universal coverage for basic health services. The paper by Mulumba et al. [7] shows that there are few spaces to discuss the inclusion of older people and people living with disabilities at the community, local or at the national level. This has translated into the breaking down of traditional family structures and the disregard of the knowledge and experience that comes from community elders, as well as a devaluing of the contributions that people with disabilities can have towards their families and the community where they live and work. This is reflected in the current post-2015 development debate, where little attention has been paid to these two growing population groups.

The realities of discrimination extend to high-income countries such as Australia, where Aboriginal and Torres Strait Islander people spoke of persistent displacement and discrimination, resulting in significant gaps in health status and life expectancy, and eroding cultural identity [8]. They recognized there is still work to be done in society in order to tackle the brutal impact of racism on their health. Yet, they also provided a glimpse of what is possible, as these communities spoke of transforming health services into ones that are grounded in Indigenous values and their own traditional structure of governance. This would offer the sense of ownership and of them being welcome.

Finally, our special issue has two commentaries. The first explores community participation and its role in the post-2015 health-related development agenda, examining the issues around effective research engagement of marginalized communities [4]. It is through meaningful participation, engaging communities and different stakeholders in binding decision-making processes that the more horizontal and inclusive approaches can begin to replace top-down processes similar to the process that gave us the original MDGs. This reflexive commentary focuses on the methodological challenges that Go4Health faced and includes experiences from our research hubs.

We conclude with a commentary by Gorik Ooms and Rachel Hammonds [9] that discusses the responsibilities that all governments face in ensuring the inalienable rights of all humans. They elaborate on the realization of social human rights through the acceptance and compliance of extra-territorial obligations that could help to transform the current international aid paradigm, turning what has routinely been seen as charity into legal obligation. They also argue that for global constitutionalism to succeed in improving fairness, human rights need to become not only more visible, but central to the development of global policy.
